# Social behavior and climate change: how rising temperatures shape insect societies

**DOI:** 10.1016/j.cois.2026.101525

**Published:** 2026-08

**Authors:** Marko Bračić, Madeleine M Ostwald, Jelena Bujan

**Affiliations:** 1 Ruđer Bošković Institute, Division for Marine and Environmental Research, Bijenička cesta 54, Zagreb 10000, Croatia; 2 Queen Mary University of London, School of Biological and Behavioural Sciences, Mile End Road, London E1 4NS, UK

## Abstract

Eusocial insects are master regulators of their thermal environments, using collective behaviors to thrive in diverse and extreme climates. Nevertheless, accelerating climate warming threatens to destabilize insect societies, due to the temperature-dependence of behaviors that underpin group organization and functioning. Here, we synthesize recent advances in our understanding of temperature effects on social behavior, focusing on the eusocial bees, wasps, ants, and termites, and scaling from individual behaviors to group-level processes. We highlight evidence that warming temperatures can disrupt foraging behavior, compromise the stability of social interactions, and exceed the capacity for nest thermoregulation. To find general patterns and understand evolutionary consequences of these effects, we need more long-term studies, research that incorporates fitness measurements, and a greater focus on tropical species, as well as understudied taxa like wasps and termites. Subtle behavioral shifts could unravel finely balanced social interactions, with cascading effects on colony fitness and ultimately ecosystem functioning.

## Highlights

• Social insects can use behavior to cope with warming, but this may be costly.
• Warming can disrupt foraging, social interactions, and development.
• Warming effects are least understood for wasps, termites, and tropical species.
• Long-term impacts of warming on colony fitness remain unclear.

**Current Opinion in Insect Science** 2026, **76**:101525

This review comes from a themed issue on **Social insects**

Edited by **Hua Yan** and **Clint Penick**

For complete overview about the section, refer “Social insects (2026)”

https://doi.org/10.1016/j.cois.2026.101525

2214–5745/© 2026 The Author(s). Published by Elsevier Inc. This is an open access article under the CC BY license (http://creativecommons.org/licenses/by/4.0/).

## Introduction

Insect declines are prevalent across the globe, and climate change is a major cause of these declines [1]. While social insects possess important adaptations for cooperatively buffering against short-term environmental change, these benefits might fail under prolonged or extreme thermal stress 2, 3. Unlike solitary insects, which can easily find a more favorable nesting site, established social insect colonies are semi-sessile, often persisting at a single location for many years and relying on central place foraging [4]. These traits that facilitate the growth of large colonies may also limit their ability to escape unfavorable environmental conditions under climate warming. How might social insect colonies mitigate these challenges as temperatures rise? Behavior is their first line of defense against thermal stress.

To understand how thermal stress affects social behavior, we review recent evidence from eusocial bees, wasps, ants, and termites (Table S1). Insect societies span a broad spectrum of obligate and facultative social behaviors [5]. In this review, we focus primarily on effects on the coordinated colony behaviors of the obligately eusocial insects, which represent the best studied insect social groups. The bulk of this research focuses on a small subset of well-studied, typically temperate-dwelling groups (especially honey bees and bumble bees), despite the high diversity of eusocial species in the tropics.

We examine how temperature influences different facets of social behavior, given its central role in regulating metabolic, developmental, and behavioral processes in ectotherms. Although our emphasis is on rising temperatures, temperature fluctuations, including unusually cool conditions, can also negatively affect eusocial insects [6]. We first consider the impact of high temperature on individual decision-making in terms of foraging activity and efficiency. We then scale up to group-level dynamics, including aggressive and protective behaviors, as well as communication among nestmates. Finally, we explore how temperature stress affects species’ extended phenotype — the nest — which can help buffer against environmental changes. We conclude by highlighting the broader consequences of these changes for the species and for species’ interactions, including mutualisms.

## Foraging

Foraging is among the most thermally stressful tasks in social insects’ repertoires, during which they must leave thermoregulated nests and perform metabolically and cognitively demanding behaviors in ambient conditions. Insects are therefore particularly vulnerable to the acute effects of heat stress while foraging, which can translate to colony-level impacts on resource intake. Warming temperatures can restrict, expand, and shift daily and seasonal foraging windows, with responses varying widely across species and local climates 7, 8, 9, 10, but see 11].

Higher temperatures can accelerate foraging activity by increasing flight performance and food intake speed in bees and wasps 12, 13, 14, running speed in ants 15, 16, and decomposition rates by termites [17]. Despite higher foraging speeds, there is growing evidence that heat stress reduces foraging effectiveness [18]. Although bumble bees flew faster under experimental heatwaves, they foraged for shorter durations, visited fewer flowers, and collected less pollen 14, 19••, 20. Larger pollen loads compound the effects of heat stress in bumble bees, increasing thoracic temperature by as much as 2°C across the range of pollen load sizes [21]. Some bees can partially mitigate this risk: nectar-loaded honey bees adjust their flight mechanics to reduce metabolic rate and increase evaporative cooling [22], while hovering bumble bees increase convective cooling by self-induced airflow [23]. Higher flight speed has also been suggested to facilitate heat loss through increased convection, although this mechanism has not been directly tested (reviewed in 6, 24••). Still, overall foraging efficiency seems to be reduced under higher temperatures due to thermal stress. Consequently, in-nest recovery between foraging bouts may be an important mechanism of thermal recovery [25].

**Figure 1:**
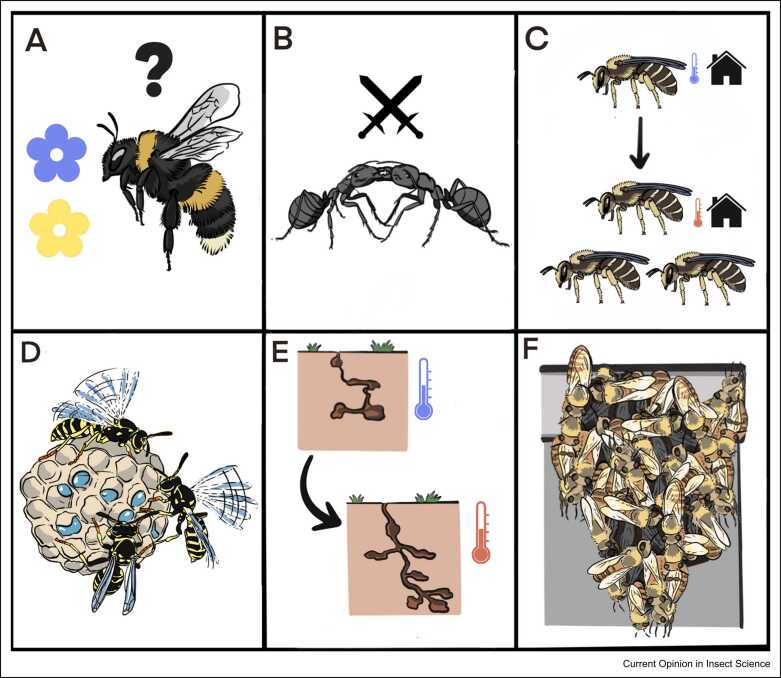
Heat stress impacts individual- and colony-level behavior in social insects, which can result in **(a)** cognitive impairment [26], **(b)** increased aggression [42], and **(c)** increased cooperative behavior [68]. Social insects can mitigate heat stress through **(d)** evaporative cooling and fanning [56], **(e)** relocation of the nest to deeper, cooler soil layers [61], and **(f)** nest evacuation or bearding [65].

Acute cognitive impacts of heat stress may impair complex navigation and memory tasks associated with foraging. Heat-stressed bumble bees perform worse at associative learning and memory tasks [26] (Figure 1a). High temperatures impair homing behavior in foraging honey bees, reducing the probability of return to the hive [27], but seem to increase homing efficiency in the temperate ant *Formica cinerea* by increasing its running speed [16]. Simultaneously, climate warming can expose brood to stressful temperatures that impair development and adult cognition, with downstream effects on foraging efficiency. Honey bees and bumble bees reared at high temperatures show deficits in short-term learning and memory as adults 28, 29. To our knowledge, similar learning experiments have not yet been done in non-flying social insects.

## Aggression, recognition, and protection

Close interactions between nestmates enable social insects to thrive across diverse habitats. By collectively directing aggression toward other colonies and species, social insects can dominate resources, raid nests, and defend their colonies. However, aggression is impacted by temperature, potentially disrupting social organization. Elevated temperatures result in increased aggression of dominant paper wasp queens toward co-foundresses [30]. Similarly, wasps (*Polybia paulista)* and trap-jaw ants (*Odontomachus chelifer*) were aggressive toward nestmates when exposed to higher temperatures (Figure 1b), potentially due to disrupted nestmate recognition linked to changes in cuticular hydrocarbons (CHCs) 31, 32. Beyond nestmate recognition, CHCs also serve a role in cuticular waterproofing [33]. Their chemical composition can be altered by temperature and humidity in wasps, ants, and bumble bees (34, 35, 36, 37, for a small elevation effect in termites see [38]). In ants, CHC alterations under dry conditions are linked to higher drought resistance 35, 39, 40, which allows them to forage in drier conditions [41]. This potential tradeoff between beneficial physiological adaptations and impaired nestmate recognition due to rapid change in CHCs could negatively affect social interactions and aggression.

In addition to impacts on nestmate aggression, temperature influences aggression between colonies and species. In ants, aggression between colonies is higher in warmer populations of a high-elevation ant (*Tetramorium alpestre*), and in ants from warmer urban populations 42•, 43. Within one ant community, temperature altered aggression toward both con- and allo-specifics, with some species becoming more and others less aggressive [44]. These results suggest that climate warming could reshape species interactions at the community level. However, other studies in ants and wasps have not detected an influence of temperature on aggression between colonies 45, 46, 47*.*

Temperature-sensitive aggression could also shape protective behavior in mutualistic relationships of social insects. For example, *Pseudomyrmex* ants defend host plants that provide extrafloral nectar and shelter, and many *Lasius* ants tend aphids that provide honeydew in exchange for protection. At higher temperatures, *Pseudomyrmex spinicola* ants were less effective at defending their bullhorn acacia host against encroaching plants [48], while four ant species were more effective at defending the whistling thorn acacia host against herbivory [49]. Under experimental warming, more ants visited pea plants but did not provide additional benefits for the plants [50]. At the community level, rising temperatures reduced the number of ant species interacting with each extrafloral-nectar-bearing plant [51]. In a cactus–ant system, species active at higher temperatures were more thermally tolerant but provided less protection, potentially reducing plant benefits and endangering these mutualisms [52]. A similar effect was observed in an ant–aphid mutualism: black garden ants (*Lasius niger)* had lower foraging efficiency, as they collected less honeydew at the highest temperature compared to the intermediate temperature, despite no decrease in ant activity or aphid honeydew production [53]. These contrasting results indicate that the effect of warming could disrupt some mutualisms, but this will depend on the specific mutualistic system.

Social insects in mutualistic relationships can also suffer from the indirect effect of warming when their partner is heat-stressed and provides fewer resources. For example, plants produced fewer floral rewards at high temperatures, which reduced bee fecundity and survival 19••, 54. Integrating information on thermal tolerances of mutualistic partners with the fitness costs imposed by rising temperatures would allow us to predict the consequences of disrupted mutualisms.

## Colony thermoregulation and development

The semi-sessile nature of eusocial insects can be a disadvantage in a changing climate, as relocating an entire colony is costly. Yet, despite these time and energy constraints, nest relocation is part of the life history of many social insects, and often occurs in response to seasonality or environmental stress [4]. Since colonies cannot always relocate quickly, they rely on behavioral and physiological strategies to reduce acute thermal stress. At the individual level, social insects, particularly the flying species, have an impressive repertoire of mechanisms to regulate their body temperature (reviewed in 6, 24••, 55). At the colony level, thermoregulation depends on coordinated social behavior. When faced with heat stress inside the nest, flying social insects will often respond with wing fanning behavior to promote convective cooling of the nest, sometimes combined with regurgitation of gut fluids and water collection to promote evaporative cooling 6, 56 (Figure 1d). However, fanning becomes ineffective when ambient temperatures are too high [28], so a paper wasp species in a warmer climate relied on it less than a species in a colder climate [56]. Evaporative cooling is a widespread thermoregulation strategy in bees and wasps (56, 57, reviewed in [28]), but there is no evidence for its use in non-flying social insects.

Colonies regulate nest temperature primarily to protect the most thermally sensitive members of the colony: its brood, including eggs, larvae, and pupae. Inability to protect brood from high temperatures can reduce egg survival, although moderate temperature increase generally speeds up development [58]. Acute heat stress might be less detrimental for ground nesters, as they can relocate brood to optimal-temperature nest areas, a task performed by specialized workers [59]. For example, during prolonged heat exposure ground-nesting ants will move the nest chambers deeper in the soil 60, 61 (Figure 1e), while fungus-growing termites (genus *Macrothermes*) adjust nest architecture to meet the thermal needs of their symbiotic fungi [62]. Thus, nest-building behavior is plastic in both ants and termites, although direct evidence of thermally induced changes in nest architecture exists only for ants.

Although nests provide protection and allow for a degree of thermoregulation, the vulnerability and protective mechanisms depend on the nest type. While ground nesters are usually well buffered from climatic extremes, above-ground nests can be exposed to direct sunlight and experience much greater climatic variability [63]. Canopy nesters have limited ways to escape rising temperatures, and this is especially true for species that nest inside cavities where movement is restricted. One strategy is brood cooling. Automated tracking of European honey bees (cavity nesters) shows collective movement away from the brood area during heatwave conditions, increasing airflow to heat-sensitive brood [64]. Honey bees can also move a subset of workers outside the nest (bearding) in response to increased temperatures (Figure 1f), but prolonged bearding impairs brood development [65]. Some canopy ant species avoid detrimental temperatures by absconding the nest with brood [66]. Both bearding and absconding are energetically costly and may compromise brood development.

Cavity nesters also show plasticity in nest choice and organization. They might be able to choose a cavity that heats more slowly, such as a live twig over a dead twig, and in so doing reduce overheating. In some cavity nesters, namely ants, a single colony can occupy multiple separated nest sites. Although this polydomy of canopy ants likely evolved as an adaptation to scarce canopy nest sites [67], it might be beneficial in a warmer world.

In addition to affecting brood development and nest thermoregulation, rising temperatures may also influence the expression of sociality and ultimately fitness. In facultatively eusocial sweat bees, longer growing seasons permit the rearing of a worker brood prior to the reproductive brood, increasing the frequency of eusocial nesting [68] (Figure 1c). Direct fitness consequences of global warming are rarely investigated, but a recent study suggested that the projected increase in temperature by 2030 could decrease male production in tropical ant species by 55% [69], which directly reduces reproductive output.

## Synthesis and outlook

Behavior is the first line of defense against thermal stress in social insects, with coordinated nest thermoregulatory behaviors offering a crucial lifeline against rising temperatures. Nevertheless, many essential colony behaviors are already disrupted under contemporary climate conditions. Foraging is limited under high temperatures due to reduced flight efficiency and cognitive impairment 26, 27. Warming can also unravel delicate social interactions, increasing nestmate aggression and reducing nestmate recognition, likely due to changes in CHC profiles that trade off with waterproofing adaptations 32, 34. Finally, warming can destabilize thermally-sensitive aggressive and mutualistic interactions between colonies and species 42•, 43, 44, 48, 49, 50. However, mutualistic interactions remain understudied and seem to respond in a context-specific manner, making it difficult to predict how warming will affect these relationships. How and when these subtle alterations in social dynamics might precipitate larger changes in colony social cohesion remains a complex, outstanding question.

Major taxonomic and geographical gaps limit our understanding of the social effects of warming. Studies of temperate honey bees and bumble bees predominate, while the effects on stingless bees, wasps, and especially termites are still largely unknown and should be a priority for future research (Table S1). Additionally, responses of tropical social insects are underexplored even though the tropics hold the majority of global biodiversity, and the most vulnerable insect taxa [70].

Due to the urgency of these questions and practical constraints of studying warming effects across the full diversity of social insects, the focus on key behavioral traits offers a powerful framework for predicting vulnerability across species. For example, ground nesting species may be better insulated against high temperatures and can further alter the nest depth and structure (Figure 1e). In contrast, cavity or canopy nesters often respond by temporarily or permanently leaving the nest (Figure 1f). Absconding from the nest with brood allows for exposure to predation, so arboreal nesting species might be at a higher risk, although this remains to be tested. Nest-cooling might be easier to perform for flying social insects, which use fanning and evaporative cooling strategies (Figure 1d), while non-flying ones rely more on other mechanisms, such as retreating to cooler microclimates. The energetic and fitness consequences of these differences have rarely been compared.

While evidence is growing for the acute effects of warming on individual behaviors, their colony-level impacts on survival, reproduction, and fitness are still unclear. Addressing these gaps requires long-term, multi-population studies that explicitly link behavioral responses to fitness consequences. Importantly, this research should determine how individual-level changes in behavior or interactions scale up to impact colony functioning. Microclimate availability and its use merit a stronger focus in future work, as an important strategy by which social insects can avoid overheating [63]. Further, while many studies have investigated the indirect impact of temperature, only a few have experimentally tested warming scenarios 19••, 25•, 64•. Manipulative studies that experimentally simulate warming offer powerful, underutilized tools for accelerating our understanding of these effects. More broadly, understanding how temperature reshapes the stability of eusocial societies while accounting for microclimate will be essential for predicting the persistence of these ecologically dominant groups in a rapidly warming world.

## Funding

MB was supported by the 10.13039/501100000780European Union as part of the NextGenerationEU program: grant NPOO.C3.2.R2-I1.06.0034. JB was funded by an ERC Starting Grant (101161952 - IGNITE-ERC-2024-STG).

## Declaration of Competing Interest

The authors declare that they have no known competing financial interests or personal relationships that could have appeared to influence the work reported in this paper.

## Data Availability

No data were used for the research described in the article.
