# Supplementary material for: Social behavior and climate change: how rising temperatures shape insect societies
Source: Curr Opin Insect Sci. 2026 Aug;76:101525. doi: 10.1016/j.cois.2026.101525 (PMC13235700; doi:10.1016/j.cois.2026.101525)
Supplement: Supplementary file 1 — Supplementary material [file mmc1.docx]

**Supplementary Information to:**

# Bračić M, Ostwald MM, Bujan J. Social behavior and climate change: how rising temperatures shape insect societies

# Table S1. Overview of reviewed studies on thermal effects in social insects, specifying taxa, study type, and studied response. The table follows the main manuscript sections, with references ordered by insect group.

| **Group** | **Taxa** | **Study type** | **Response** | **Reference** |
| --- | --- | --- | --- | --- |
| **Section: Foraging** | | | | |
| Bee | Three bee taxa | Field | Visitation rate to flowers | [10] Jaboor et al. 2022 |
| Bee | Bumblebee, *Bombus terrestris* | Lab | Flight performance | [13] Kenna et al. 2021 |
| Bee | Bumblebee, *Bombus terrestris* | Lab | Foraging performance & metabolic rate | [14] Gérard et al. 2024 |
| Bee | Bumblebee, *Bombus impatiens* | Lab | Foraging performance & nectar intake | [18] Hemberger et al. 2023 |
| Bee | Bumblebee, *Bombus eximius* | Lab | Foraging, energy budget, gene expression | [19] Kuo et al. 2023 |
| Bee | Bumblebee, *Bombus impatiens* | Field | Pollen load | [20] Naumchik & Youngsteadt 2023 |
| Bee | Bumblebee, *Bombus impatiens* | Lab | Thermal recovery & heat tolerance | [21] Quinlan et al. 2023 |
| Bee | Honey bee, *Apis mellifera* | Lab | Flight mechanics & heat tolerance | [22] Glass et al. 2024 |
| Bee | Bumblebee, *Bombus* sp. | Lab | Convective cooling during flight | [23] Glass et al. 2026 |
| Bee | Bumblebee, *Bombus impatiens* | Lab | Thermal recovery & heat tolerance | [25] Quinlan et al. 2023 |
| Bee | Bumblebee, *Bombus terrestris* | Lab | Learning & memory | [26] Gérard et al. 2022 |
| Bee | Honey bee, *Apis mellifera* | Lab | Homing behavior | [27] DesJardins et al. 2024 |
| Bee | Bumblebee, *Bombus terrestris* | Lab | Sensory responsiveness & morphology | [29] Perl et al. 2022 |
| Wasp | Yellowjacket, *Vespula germanica* | Field | Foraging activity | [7] Kasper et al. 2008 |
| Wasp | Yellowjacket, *Vespula germanica* | Field | Foraging efficiency | [12] Kovac et al. 2018 |
| Ant | Desert ants, *Cataglyphis* spp. & *Ocymyrmex* spp. | Field | Daily & seasonal foraging activity | [8] Wehner & Wehner 2011 |
| Ant | Five ant species | Field | Foraging at bait & thermal tolerance | [11] Youngsteadt et al. 2023 |
| Ant | Harvester ant, *Pogonomyrmex barbatus* | Field | Foraging behavior & thermal tolerance | [15] Roeder et al. 2022 |
| Ant | Wood ant*, Formica cinerea* | Field | Running speed & homing | [16] Ślipiński & Cerdá 2022 |
| Ant | Three species of cavity-nesting ants | Field | Thermal tolerance, foraging & defense | [43] Harris et al. 2024 |
| Termite | African savanna termite community | Field | Seasonal activity | [9] Davies et al. 2015 |
| Termite | Multiple termite species | Modelling | Wood decomposition | [17] Zanne et al. 2022 |
| **Section: Aggression, recognition, and protection** | | | | |
| Bee | Bumblebee*, Bombus lapidarius* | Field | CHC profile & morphology | [36] Straub et al. 2022 |
| Bee | Bumblebee, *Bombus lucorum* | Field | CHC profiles | [37] Maihoff et al. 2023 |
| Bee | Solitary bee, *Osmia lignaria* | Lab | Indirect effects: fecundity & survival | [54] Walters et al. 2024 |
| Wasp | Paper wasp, *Polistes dominulus* | Lab | Aggression & group stability | [30] Tibbetts & Reeve 2008 |
| Wasp | Wasp, *Polybia paulista* | Lab | Nestmate recognition & aggression | [31] Michelutti et al. 2022 |
| Wasp | Three species of *Polistes*  & *Polybia* wasps | Lab | Survival & CHCs | [34] Michelutti et al. 2018 |
| Wasp | Yellowjackets*, Vespula vulgaris* & *V. germanica* | Field | Colony-level aggression | [46] Jandt et al. 2020 |
| Wasp | *Yellowjackets, Vespula vulgaris* | Field | Defensive behavior | [47] Detoni et al. 2023 |
| Ant | Trap-jaw ant, *Odontomachus chelifer* | Lab | Nestmate recognition & aggression | [32] Silva et al. 2024 |
| Ant | Three ant species | Lab | CHC acclimation | [35] Baumgart et al. 2022 |
| Ant | Cavity-nesting ants, *Temnothorax longispinosus* & *T. ambiguus* | Field | CHC acclimation & resistance | [39] Menzel et al. 2018 |
| Ant | Harvester ant*, Pogonomyrmex californicus* | Lab | CHC, resistance & body mass | [40] Ostwald et al. 2023 |
| Ant | Desert ant,  *Pogonomyrmex barbatus* | Field | CHC & collective response to drought | [41] Menzel et al. 2026 |
| Ant | Ant, *Tetramorium alpestre* | Field | Aggression among workers | [42] Krapf et al. 2023 |
| Ant | Three ant species | Field | Exploration & aggression | [44] Menges et al. 2024 |
| Ant | Cavity-nesting ant, *Temnothorax longispinosus* | Lab | Behavioral syndromes | [45] Segev et al. 2017 |
| Ant | Thorn-nesting ant, *Pseudomyrmex spinicola* – acacia plant | Field | Mutualistic defense | [48] Shastri et al. 2025 |
| Ant | Multiple ant species–  acacia plant | Field | Mutualistic defense | [49] Tamashiro et al. 2019 |
| Ant | Multiple ant species– partridge pea plant | Field | Presence of ants on mutualist plant | [50] Magnoli et al. 2023 |
| Ant | Many forest ant–plant species | Field | Presence of ants on mutualist plant | [51] Câmara et al. 2024 |
| Ant | Multiple ant species –– cactus | Field | Presence of ants on mutualist plant | [52] Fitzpatrick et al. 2014 |
| Ant | Black garden ant, *Lasius niger* – aphid *Aphis fabae* | Field | Mutualistic interactions | [53] Blanchard et al. 2021 |
| Termite | Termite, *Nasutitermes coxipoensis* | Field | Cuticular profile & aggression | [38] Camarota et al. 2025 |
| **Section: Colony thermoregulation and development** | | | | |
| Bee | Honey bee, *Apis mellifera* | Field | Water collection & storage | [57] Ostwald et al. 2016 |
| Bee | Honey bee, *Apis mellifera* | Field | Activity & spatial movement | [64] Jhawar et al. 2023 |
| Bee | Honey bee, *Apis mellifera* | Field | Bearding responses | [65] Rowe et al. 2025 |
| Bee | Sweat bee, *Halictus rubicundus* | Field | Foraging & projected nesting | [68] Schürch et al. 2016 |
| Wasp | Paper wasps, *Polistes biglumis* & *P. gallicus* | Field | Nest & body thermoregulation | [56] Stabentheiner et al. 2022 |
| Ant | Argentine ant, *Linepithema humile* | Lab | Development time & survival | [58] Abril et al. 2010 |
| Ant | Carpenter ant*,  Camponotus floridanus* | Lab | Brood transport & task specialization | [59] McGregor et al. 2024 |
| Ant | Ant, *Formica podzolica* | Lab | Nest architecture | [60] Sankovitz & Purcell 2021 |
| Ant | Black garden ant, *Lasius niger* | Field | Nest architecture & soil turbation | [61] García Ibarra et al. 2024 |
| Ant | Four twig-nesting ant species | Lab | Evacuation under heat stress | [66] Bujan & Yanoviak 2022 |
| Ant | Forest canopy ant community | Field | Connectivity & colonization | [67] Adams & Schnitzer 2019 |
| Ant | Tropical ant community | Field | Male production | [69] Uquillas et al. 2025 |
| Termite | Mound-building termite*, Macrotermes* – *Termitomyces* fungi | Field | Nest architecture & fungal symbionts | [62] Vesala et al. 2019 |

Notes: Study type was classified based on where the insects lived. Reference numbers correspond to the reference list in the main manuscript.
